# Does Environmental Walkability Matter? The Role of Walkable Environment in Active Commuting

**DOI:** 10.3390/ijerph17041261

**Published:** 2020-02-15

**Authors:** Eun Jung Kim, Jiyeong Kim, Hyunjung Kim

**Affiliations:** 1 Department of Urban Planning, Keimyung University, 1095 Dalgubeol-daero, Dalseo-gu, Daegu 42601, Korea; kimej@kmu.ac.kr (E.J.K.); th154@naver.com (J.K.); 2 Department of Civil and Environmental Engineering, Seoul National University, Gwanak-ro 1, Gwanak-gu, Seoul 08826, Korea

**Keywords:** active commuting, walking, cycling, Walk Score, Walkability Score, multilevel logistic regression model, geographic information system (GIS), Seoul

## Abstract

Since walkability plays an important role in active commuting, several cities are actively promoting its integration into urban and environmental planning policies. This study examined the association between walkability and active commuting in Seoul, Korea. A multilevel logistic regression model was used to examine the correlation between Walkability Score and the probability of active commuting after controlling for individual variables. The analysis used 129,044 individual samples nested within 424 administrative districts (dongs). In this study, three models were tested: Model 1 contained only individual variables, Model 2 contained individual variables and Walkability Score, and Model 3 included neighborhood-level variables in addition to the variables of Model 2. The results showed that the Walkability Score was significantly correlated with the odds of active commuting. Specifically, every additional one-point increase in Walkability Score was associated with 0.3% higher odds of active commuting (Model 2: odds ratio (OR) = 1.003, 95% confidence interval (CI) = 1.001–1.005; Model 3: OR = 1.003, 95% CI = 1.001–1.006). Additionally, public transportation density was also positively correlated with the odds of active commuting. The odds of active commuting were positively correlated with younger age, female, lower-income, and having no car. Based on the findings, policy recommendations in urban planning and design, transport engineering, and environmental planning are provided.

## 1. Introduction

Active commuting (e.g., walking and cycling to work or school) has numerous advantages. It is a cost-effective transportation mode, being an affordable means of transportation with little maintenance required [1]. Reduced car use due to active commuting can lead to a decrease in air pollution and traffic congestion, and more efficient use of urban land due to a reduction in parking lots [2,3,4]. Moreover, active commuting has several health benefits, including increasing physical activity [5] and promoting mental health [6,7,8], and reducing obesity [9], cardiovascular diseases [10,11,12], type 2 diabetes [13], cancers [10,12], and all-cause mortality [10,14,15]. Avila-Palencia et al. have examined the relationship between bicycle commuters and perceived stress in Barcelona, Spain. After controlling for individual and environmental variables, their study found that bicycle commuters who commuted by bicycle for more than four days a week were less stressed than non-bicycle commuters [6]. Avila-Palencia extended her study with colleagues to test transport mode and mental health and social cohesion in seven European cities. She found that walking was associated with good self-perceived health, higher vitality, and more frequent contact with family/friends, and cycling was correlated with good self-perceived health, higher vitality, less perceived stress and loneliness [7]. Likewise, a study conducted by Zijlema et al. has examined the association between active commuting through natural environments and mental health. They collected data using questionnaires from Spain, the Netherlands, Lithuania, and the United Kingdom, and used multilevel models at the city and neighborhood levels. They found a strong association between active commuting through natural environments and mental health [8]. 

Several studies have used health outcome variables rather than mental health measures as health indicators. Celis-Morales et al. examined the relationship between active commuting and cardiovascular disease, cancer, and all-cause mortality. They used data from UK Biobank, which was recruited from 22 sites across the UK. They found that active commuting was associated with a lower risk of cardiovascular diseases, cancer, and mortality [10]. Blond and Rasmussen with their colleagues focused on the effects of cycling on health promotion and conducted two empirical studies. They conducted their studies in Danish adults and collected data from the prospective cohort study, “Diet, Cancer, and Health”. They found that cycling was associated with lower risks of coronary heart disease [11] and type 2 diabetes [13]. Andersen and colleagues have also focused on cycling as active commuting in Copenhagen, Denmark, and found cycling to work was associated with a lower risk of mortality [14]. 

Meanwhile, there have been studies that calculated the benefits of active commuting in monetary values. One report from Public Health England estimated that the economic cost of physical inactivity to the National Health Service in England is more than ₤450 million (approximately US $586 million) a year [16]. The British government has also reported that achieving their policy targets for walking and cycling could save ₤567 million (approximately US $740 million) a year on air quality [17]. 

Seoul is the most urbanized city in Korea. It has chronic urban problems typical of several megacities, such as long commuting distance and times, heavy traffic congestion, and severe air pollution, which are mainly caused by high levels of car dependence. The active commuting (e.g., walking and cycling) of Seoul was 28.0% in 2015 of the total commuting trip [18,19], which indicates the majority of commuting in Seoul is dependent on motorized commuting. This is causing societal costs in various areas, including environmental and public health, as discussed earlier. To address this, the city of Seoul recently proposed a number of urban policies to promote walking and cycling. Specifically, Seoul suggested a project titled, “Walkable City, Seoul” and “Two-wheel Seoul”, whose main objective is to create a safe and convenient walking and cycling environment not only for reducing social costs by decreasing the number of cars but also to enhance citizens’ health. A lot of projects have been promoted since 2016 including “Safe Walking Street for Children” to expend Children Protection Zone and Speeding Warning Signs, and a “Road Diet” project to reduce roadway and expanding the pedestrian road. Also, the city of Seoul is creating a pedestrian-oriented traffic environment thorough maintaining and connecting pedestrian roads. To promote cycling, Seoul is expanding bike-related infrastructure such as cycle lanes and bike-rental stations, and also operating Seoul’s public bicycle sharing system, “Ddareungi”, to encourage the greater use of bikes as a means of transportation [20]. 

It is often said that the neighborhood environment is important for promoting active commuting. Some evidence suggests that the built environment is associated with walking and cycling for transportation. A higher level of active commuting is associated with higher levels of street connectivity, population density, mixed land use, and public transportation density [21,22,23,24,25,26]. More specifically, walkability is positively correlated with walking and cycling by commuters. The walkable environment can help encourage people who commute short distances to walk or cycle. The walkable environment also induces people who commute using public transport to walk or cycle to where they access transportation (e.g., bus stops and subway stations) [25,27,28]. 

Numerous studies have focused on the measurement of environmental walkability, using objectively measured indices in particular [29,30,31,32,33,34,35,36]. These indices are all measured using geographic information systems (GIS), and the typical ones are Walkability Index, Moveability Index, Walk Score, Pedshed, and Pedestrian Environment Index. The Walkability Index included elements such as residential density, retail floor area ratio, street connectivity, and land-use mix [33,35]. Moveability Index derived from the concept of the Walkability Index, an index that has been widely used in recent years. This includes elements of street connectivity, destination density, and the level of urbanization [31,32]. Pedshed is another index of walkability for the pedestrian catchment, calculated from the network/Euclidean buffer ratio [30]. The Pedestrian Environment Index is a newly developed index to measure pedestrian friendliness, calculated with four components including land-use mix, population density, commercial density, and intersection density [36]. 

One such index, Walk Score, is a popular measure which several empirical studies have used [37,38,39,40]. The Walk Score calculates access to nine types of utilitarian destinations including grocery stores, restaurants, shopping centers, coffee shops, banks, parks, schools, bookstores, and places of entertainment in a given area. In addition, the area may receive a penalty for having poor pedestrian friendliness (e.g., intersection density and average block length). The final score ranges from 0 (car-dependent) to 100 (walker’s paradise) [41]. The Walk Score has the disadvantage of mainly measuring the accessibility of amenities and not measuring the qualitative factors of the urban environment. Therefore, some studies have conducted analyses comparing the Walk Score to other indices to verify its validity and reliability, and found that the Walk Score is suitable for use as a measure of environmental walkability [39,42].

A growing body of literature has found that Walk Score had positive associations with walking in communities [43,44,45,46,47,48]. Most of these studies have been conducted in the United States and Canada, where Walk Score data is available [49]. Some studies have been conducted to develop data in areas where no data is currently available on the Walk Score website [50]. Reyer et al. assessed the level of walkability in Stuttgart, Germany, using the Walk Score algorithm. They found a significantly positive relationship between Walk Score and active transportation (e.g., the walked distance for transport per week, the minutes of walking for transport per week, and the number of walking trips per week) after controlling for socioeconomic factors at the individual level [48]. Recently, Kim et al. also assessed the walkability level of Seoul, Korea by adapting the Walk Score methodology [51]. They found a positive association between Walk Score (in their study, “Walkability Score”) and pedestrian satisfaction. 

As in the empirical studies of the United States, Canada, and Germany [43,44,45,46,47,48], do people who live in areas with a higher level of walkability walk and bike more than those who live in areas with a lower level of walkability in Seoul, Korea? There are some previous studies from Asian countries similar to this topic, however, most focused on either promoting physical activity for older adults [52,53,54,55], or using survey/interview-based measurement of walkable environments [56,57]. Most importantly, there has been little research on the association between walkable environments and active commuting. Like Seoul, many Asian cities are in highly dense urban form, and most of the population is concentrated in big cities which leads to lacking space for physical activity. Especially for highly dense cities, promoting active commuting could be a realistic way to prevent physical inactivity. However, objectively measured indices measuring the level of walkability based on the built environment are mostly developed from Western countries, therefore works in Asian countries are lacking in spite of the necessity of investigation. Therefore, we consider our research question is timely and appropriate as the importance of walkable neighborhood environments on promoting a daily based physical activity is arising in literature. The purpose of this study is to examine if the walkable environment correlates with active commuting (e.g., walking and cycling) in Seoul. It also acts as a validation check on the applicability of the Walkability Score for assessing the level of neighborhood walkability in Seoul. In this study, the following hypothesis is tested: *Areas with more walkable environments have correlation with higher odds of walking and cycling to work and school (e.g., active commuting) than areas with less walkable environments.*

## 2. Materials and Methods 

### 2.1. Study Area 

Seoul is the capital of Korea, located in the northwest of the country. It has the highest population density (16,364 persons/km^2^) in Korea [58]. Therefore, solving its transportation problems including traffic congestion, air pollution due to heavy traffic is of primary importance. Recently, the city of Seoul has been heavily promoting policies to encourage walking and cycling as one of the city’s major issues under the project called “Walkable City, Seoul” [20]. Meanwhile, a recent study has measured the Walkability Score in Seoul [51], while there have been no such cities that have assessed the walkability levels in Korea yet. In other words, since there exists a walkability dataset for Seoul, it is straightforward to conduct research on the city. In addition, it has been more than 3 years since the city of Seoul promoted “Walkable City, Seoul” as one of the major policies, however, there is little research on the correlation between environmental walkability and active commuting in Seoul.

### 2.2. Variables: Data

#### 2.2.1. Individual-Level Variables

● Outcome measure: travel mode of commuting (non-motorized /motorized commuting)

This study used the ‘travel mode to work and school’ data from the 2016 Household Travel Diary Survey of the Korea Transport Database as its dependent variable (Table 2). This mode includes commuting transport and is extracted by home-based origin or destination [59]. The Household Travel Diary Survey defines four types of travel purpose: work, school, shopping, and leisure. Only the cases of travel to work and school were extracted, which include walking, cycling, public transit (e.g., bus, subway), and private automobile. This study excluded the cases of a commuter using multiple modes in a trip. We dichotomized the variable into 1 for non-motorized transport (“walking” and “cycling”) and 0 for motorized transport (“public transit” and “private automobile”). In total, 129,044 commuter trips were analyzed. Approximately 44.0% of respondents took walking and cycling trips for commuting purposes while the remaining 56.0% used public transport and private automobiles.

● Socioeconomic status

Individual variables included were age, gender (male/female), income level, and car ownership (no/yes), also sourced from the 2016 Household Travel Diary Survey [59]. The mean age was 37.6 (standard deviation (SD) = 15.5) and the mean income level was 4.0, which equates to a monthly income of 3–5 million won (approximately US $2727–4545). Approximately 56.3% of the respondents were male (female: 43.7%), and 71.9% of respondents owned a private automobile. 

#### 2.2.2. Neighborhood-Level (Dong-Level) Variables

● Walkability Score (key independent variable)

This study used the Walkability Score from Kim et al. [51] as an independent variable, which measures Seoul’s walkability level using the Walk Score algorithm. The Walkability Score was derived by dividing the entirety of Seoul, except for the greenbelt and the Han River, into 100 × 100 m grid cells and calculating the score for a center point of each grid cell. As shown in Table 1, the Walkability Score is derived by considering accessibility to nine amenity facilities and pedestrian friendliness. Information on details such as the calculation method and descriptive statistics of outputs can be found in the study by Kim et al. [51]. 

**Figure 1:**
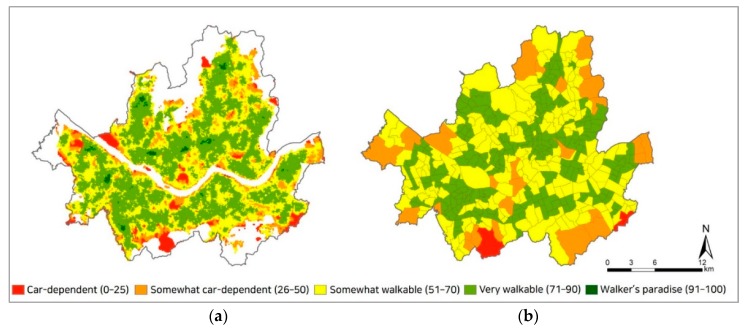
Walkability Score of Seoul: (**a**) Walkability Score at grid point-level (N = 44,000; Source: [51]); (**b**) Walkability Score at dong level (N = 424); Areas marked with null values in Figure 1a (greenbelt and Han River) are not included when calculating the Walkability Score for each dong in Figure 1b.

Seoul consists of 424 administrative districts (“dong” in Korean), which are used as the neighborhood-level units in this study. The Walkability Score, measured at the grid point-level (N = 44,000), was then aggregated at the dong level (N = 424) for analysis (Figure 1). The mean Walkability Score of the 424 dongs was 68.2 (SD = 9.8; Table 2).

● Additional neighborhood environmental factors

Additional environmental variables analyzed include land use mix, residential density, and public transport density. Some studies have found that land-use mix is significantly associated with walking for transport [26,67]. Therefore, this study included the land-use mix variable that measures the mixture of residential, commercial, industrial, and greenspace. It is measured by an entropy index ranging from 0 to 1, where 0 is single land use and 1 is perfect mixing within the land-use types. Residential density (i.e., number of residential units per square meter) is considered an environmental factor because it is the most commonly used index to measure urban form that is correlated with walking and physical activity [26,68,69,70,71]. Public transport use has also been found to contribute to enhanced physical activity [25]. Therefore, public transport destination density (i.e., the number of bus stops and subway stations per square kilometer) was also included as an additional neighborhood environmental factor. Since the density of Seoul is very high, a lot of traffic occurs especially during commuting. Therefore, the city of Seoul has built a very tightly knitted public transportation network in and between bus and subway, and since 2004, the transfer fee between public transportations (e.g., subway to bus, bus to subway) is free of charge [72]. This had led to results in the use of public transportation trips in Seoul; a combination of buses and subways. Therefore, in this study, we calculated public transport density by combining bus stops and subway stations. All environmental variable data were collected in 2017, measured at dong level, and captured using ArcGIS 10.6 (ESRI, Redlands, CA, USA).

### 2.3. Analytical Methods

Bivariate analysis was used to compare the differences between variables at the individual level and neighborhood level by Walkability Score. Specifically, the *t*-test was employed for comparison of travel mode types and urban forms between areas with higher walkability and areas with lower walkability. A multilevel logistic model was used to examine the probability of non-motorized commuting transport (e.g., walking and cycling for commuting trips) by considering both individual-level and neighborhood-level (dong-level) variables. The analysis includes 129,044 individual commuters nested within 424 dongs using a two-level structure (individual: level 1, dong: level 2). Two-level logistic regression analysis was performed using R (version 3.6.1).

## 3. Results

### 3.1. Bivariate Analyses of Walkability Score and Individual-Level and Neighborhood-Level Variables

#### 3.1.1. Travel Mode Comparison between Groups by Level of Walkability Score

**Figure 2:**
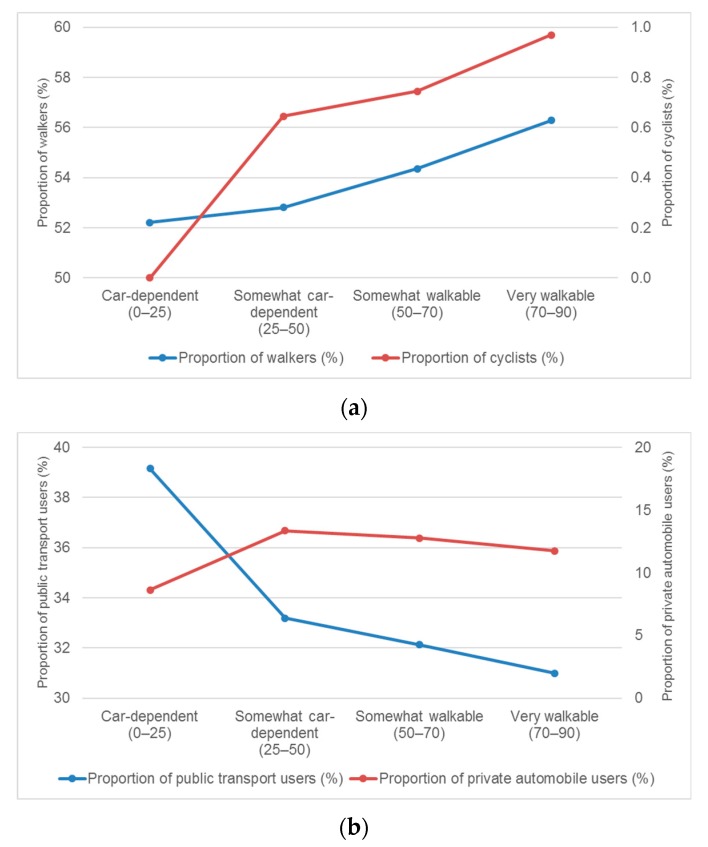
The proportion of (**a**) non-motorized commuters; (**b**) motorized commuters.

Figure 2 shows the proportion of non-motorized (walking and cycling) and motorized (public transport and private automobile) commuters by the level of Walkability Score in Seoul. As shown in Figure 2a, 56.3% of residents were walkers in very walkable areas, whereas only 52.2% were walkers in car-dependent areas. Similar to this, almost 1.0% of people used a bicycle for commuting in very walkable areas, while nobody (0.0%) cycled in car-dependent areas. The figure shows that the higher the Walkability Score, the higher the proportion of people walking and cycling for commuting purposes. 

Conversely, Figure 2b illustrates that the proportion of public transit users in areas with higher Walkability Score is considerably less than in car-dependent areas. The proportion of public transit users dramatically decreased from 39.2% in car-dependent areas to 31.0% in very walkable areas. For the proportion of private automobile users, an increase from 8.6% in car-dependent areas to 13.3% in somewhat car-dependent areas, followed by a general decline to 11.7% in very walkable areas was found.

#### 3.1.2. *t*-Test for the Comparison between Areas with High Walkability and Low Walkability Scores

As shown in Figure 2, higher proportions of walkers and cyclists in areas with higher Walkability Scores were found than in areas with lower Walkability Scores. However, these results were not statistically significant. Therefore, the mean difference between areas with higher walkability and lower walkability are compared. 

To do so, we classified the entirety of Seoul as either more walkable or less walkable. More walkable areas were those with a Walkability Score ≥ 50 (i.e., somewhat walkable and very walkable); less walkable areas were those with a Walkability Score < 50 (i.e., car-dependent and somewhat car-dependent). We then calculated the mean value for each dong. Of the 424 dongs, 395 had a mean Walkability Score of 50 or more, and 29 dongs had a mean Walkability Score of less than 50. 

Table 3 shows the results of the *t*-test between the two groups (high Walkability Score vs low Walkability Score). There were significantly more walking trips for commuting purposes in the more walkable areas (55.4%) than those with a less walkable environment (50.5%) at the 0.05 significance level. However, the other three travel modes (cycling, public transport, and private automobile) had no statistical mean differences between areas with high and low Walkability Scores. 

Urban form variables of residential density and public transport density in the areas with a more walkable environment (Walkability Score ≥ 50) were significantly higher than those with a less walkable environment (Walkability Score < 50). This is consistent with the results of previous studies [25,26,68,69,70,71]. With respect to land use mix, this study found contrasting results with those of other studies [26,67]. Although proper land-use mix is generally known to increase the level of active transport (e.g., walking and cycling), there is some contrary evidence suggesting that mixed-use development in some high-density cities may not always produce a positive effect on walking and cycling activities [75,76,77]. This is because high-density cities would reduce the chance for people to undertake walking or cycling as people could move easily to another neighborhood by public transportation [75,77]. Seoul is one the high-density cities; while the total area of Seoul constitutes 0.60% of Korea, the total population was almost 19.04% (the total population of Seoul was 9,857,426) of the national population as of 2017 [78], and having a good public transportation in the neighborhood. This might be applied to Seoul, and our finding could contribute to this contrary evidence showing the areas with higher Walkability Score had lower level of land use mix.

### 3.2. Multilevel Analysis for Predictor of Non-Motorized Commuting Trips

To investigate the role of the walkable environment on the incidence of active commuting trips, we employed a multilevel logistic regression model using two levels of data: individual commuter (level 1) and dong (level 2). A total of 129,044 individuals nested within 424 dongs were used. Individuals living in the same area (dong) share the same environmental conditions. 

The results of the multilevel logistic regression model for the odds of non-motorized (walking and cycling) commuting are presented in Table 4. Model 1 is the unconditional model with only individual-level predictors, while models of 2 and 3 include neighborhood-level variables. As a neighborhood-level variable, only the Walkability Score is considered in Model 2, and additional urban form variables, as well as the Walkability Score, are included in Model 3. The Intraclass Correlation Coefficient (ICC) shows that 2.1% of the total variance was at the neighborhood level from models 2 and 3. Based on Akaike Information Criterion (AIC) and Bayesian Information Criterion (BIC) values, Model 3 is preferred. 

The findings of the analyses are three-fold. First, the neighborhood-level Walkability Score had a significantly positive relationship with the individual-level odds of non-motorized trips at the 0.01 significance level. People who lived in areas (dongs) with a higher Walkability Score were much more likely to undertake walking and cycling trips for commuting purposes. Specifically, every additional one-point increase in Walkability Score was associated with 0.3% higher odds of walking and cycling trips for commuting purposes from both Model 2 and Model 3 (Model 2: odds ratio (OR) = 1.003, 95% confidence interval (CI) = 1.001–1.005; Model 3: OR = 1.003, 95% CI = 1.001–1.006). This result empirically supports the hypothesis that areas with more walkable environments have correlation with higher odds of walking and cycling to work and school (i.e., active commuting) more than areas with a less walkable environment.

Second, public transport density was positively correlated with the odds of non-motorized trips at the 0.05 significance level (OR = 1.012, CI = 1.001–1.023). This result is similar to that of previous studies, which found that access to public transportation was positively correlated with walking and cycling [25,27,28]. Other neighborhood-level variables had no statistical relationship with the odds of non-motorized trips from Model 3. 

Third, the effects of all individual variables are fairly consistent across all models. The odds of non-motorized transport were positively correlated with the female gender (reference group: male), while it was negatively associated with age, level of income, and car ownership (reference group: no). Thus, those who were female, younger, on a lower income, and had no car were more likely to walk and bike for commuting purposes.

## 4. Discussion

Encouraging active commuting is one of the most important agendas in the fields of urban planning and design, transportation planning and engineering, environmental planning, and even public health. Several studies have identified individual health benefits of active commuting [5,6,7,8,9,10,11,12,13,14,15], and a report from Public Health England also identified its benefits for the wider population including reductions in air pollution, noise, and economic costs [79]. Thus, efforts are being made by policymakers from a multitude of countries to change citizens’ commuting behaviors toward active commuting, and Seoul is no exception [20]. 

The quality of the environment is one of the most important factors influencing the level of walking and cycling taking place in a city. Therefore, this study examined the role of environmental walkability on active commuting. Specifically, it tested the hypothesis that areas with more walkable environments have correlation with higher odds of walking and cycling to work and school (i.e., active commuting) more than areas with less walkable environments. 

In this study, we found that the level of walkability was positively correlated with the probability of walking and cycling for commuting purposes in Seoul. This finding supports the hypothesis that the walkable environment has a strong role in the incidence of active commuting. Second, public transportation density had a significantly positive relationship with the probability of walking and cycling. This indicates that public transportation destinations, including subway stations and bus stops, encourages walking and cycling. As discussed earlier, some studies have shown that easy access to public transportation leads people to walk and bike to public transport facilities [25,27,28]. In other words, more people can walk and bike in areas with a higher density of public transportation. Third, other individual variables were correlated with active commuting, which is consistent with the results of previous empirical studies [39,80]. Individual factors such as age, gender, income, and car ownership were strongly correlated with active commuting. The odds of active commuting were strongly associated with younger age, female, lower income, and having no car.

Based on the findings of this study, some suggestions for future policymaking can be made. First, creating walkable environments could be considered a measure for sustainable development indicators within local and national urban and environmental policies. Policy actions to promote active commuting will directly contribute to achieving the third goal of the United Nations 2030 Sustainable Development Goals (SDGs), i.e., good health and well-being [81]. Creating a walkable environment could be tied to the SDGs and put forward as a city-wide policy. Second, neighborhood- and city-level efforts seem necessary for encouraging active commuting. Our findings clearly indicate that the level of neighborhood walkability can play an important role in promoting walking and cycling in daily life. Actions by the city government of Seoul should include creating its own customized strategies in order to more effectively promote active commuting. The city of Seoul should earmark investments for building walkable and cyclable environments. In particular, since Seoul Metropolitan Government is promoting the “Walkable City, Seoul” project as one of the major projects for urban planning and design, the city of Seoul is creating a pedestrian-oriented traffic environment to maintain pedestrian roads and form pedestrian walkways that connect to one another by linking the disconnected pedestrian paths [20]. From our findings, the city of Seoul can consider maintaining and connecting pedestrian pathways not only for leisure walking but also for active commuting in terms of promoting citizens’ daily physical activities.

This study has several limitations, and directions for future study to address them are as follows. First, although this study could examine the overall correlation between walkability and active commuting throughout the entire Seoul, it could not include different characteristics by sub-regions. In further research, the areas with low levels of walkability and/or active commuting in particular should be examined. Second, this study dichotomized travel purpose into motorized and non-motorized transport for the use of a multilevel logistic model. For this reason, it was somewhat difficult to find and discuss the results for each transportation purpose. In a future study, it may be necessary to use a multilevel analysis for categorical outcomes. Third, the study only examined the correlation between walkability and active commuting. Further research could go beyond active commuting to empirically analyze how environmental walkability plays a role in reducing diseases (e.g., obesity, cardiovascular disease, and type 2 diabetes) and in improving health conditions (e.g., self-reported health status and mental health). Fourth, the Walkability Score used was adapted from the popular Walk Score index; however, walkability level using another objectively measured index can be considered. According to previous empirical literature on Seoul, other indices such as Walkability Index, Pedshed, and Moveability Index could be examined [42]. Lastly, the Walkability Score used in this study does not include elements that evaluate the quality of the environment. The behavior of walking and cycling depends on the quality of the environment (e.g., the level of greenness of parks, the quality of pedestrian streets, etc.). For example, some studies found that cognitive perception of urban form or urban design qualities is important for pedestrians [82,83]. In future studies, it is necessary to use a walkability index that considers variables related to environmental quality. 

Despite these limitations, this study has the following implications. First, we used a relatively large amount of data including 129,044 covering all administrative units (N = 424) in Seoul. Therefore, the study was able to meaningfully look at the overall associations between active commuting and environmental walkability in Seoul. Second, we employed an objectively measured environmental walkability index, which can more reliably stand for environmental walkability, and thus be more useful for policy purposes, than a subjective index. Third, because the correlation of environmental walkability and active commuting was analyzed using a multilevel logistic model, factors from both individual and neighborhood levels could be taken into account. Furthermore, because individual-level variables were nested within the neighborhood, the multilevel analysis allowed the impact of multilevel factors on the dependent variable (active commuting) to be examined. Lastly, this study empirically investigated the relationship between environmental walkability and active commuting in Seoul. Although it has been several years since the city of Seoul implemented the “Walkable City, Seoul” project, no studies have examined how the creation of walkable environment correlates with walking and cycling. In this aspect, we believe this study is significant in that we explored the relationship between the level of neighborhood walking environment and walking and cycling in Seoul.

## 5. Conclusions

This study found a positive correlation between objectively-measured environmental walkability and active commuting outcomes using a multilevel logistic regression model in a Korean context. In general, personal behavior variables such as walking and cycling are highly influenced by individual characteristics (e.g., gender, age, income, etc.). Nevertheless, this study found a significant correlation between Walkability Score and the odds of active commuting. Based on the results of this study, we can expect several related policies to promote active commuting in the fields of urban regeneration, environmental planning, and transportation engineering. Furthermore, it is expected that health-promotion policies would benefit from the encouragement of active commuting. 

## Figures and Tables

**Table 1:** Categories, measures, and data source for the Walkability Score calculation.

Category	Measure	Data Source
Distance to amenity	Grocery	Seoul Open Data Plaza [60]
	Restaurants	
	Shopping	Road Name Address [61]
	Coffee	Seoul Open Data Plaza [60]
	Banks	National Spatial Data Infrastructure Portal [62]
	Parks	Road Name Address [61]
	Schools	
	Books	
	Entertainment	Website of three major cinemas [63,64,65], Open Data Portal [66], and Road Name Address [61]
Pedestrian Friendliness	Intersection density	National Spatial Data Infrastructure Portal [62]
	Average block length	

The original contents of this table are came from Walk Score methodology [41] and Table 1 of Kim et al. [51], and they were newly edited here.

**Table 2:** Measurement, data source, and descriptive statistics of variables.

Variable	Measurement	Data Source	Frequency (%)	Mean (SD)
Dependent Variable				
Travel mode to work and school	Binary: 0 = Motorized commuting (public transport and private automobile), 1 = Non-motorized commuting (walking and cycling)	Household Travel Diary Survey from the Korea Transport Database [59]	56,724 (44.0%) 72,320 (56.0%)	
Individual Variables (Level 1)				
Age	Continuous: Age	Household Travel Diary Survey from the Korea Transport Database [59]		37.6 (15.5)
Gender	Binary: 0 = male, 1 = female		72,638 (56.3%) 56,406 (43.7%)	
Income	Continuous: 1 = less than 1 million won, 2 = 1–2 million won, 3 = 2–3 million won, 4 = 3–5 million won, 5 = 5–10 million won, 6 = more than 10 million won			4.0 (1.0)
Car ownership	Binary: 0 = no, 1 = yes		36,321 (28.1%) 92,723 (71.9%)	
Neighborhood Variables (Level 2; Dong level)				
Walkability Score	Continuous: Walkability Score	Kim et al. [51]		68.2 (9.8)
Land use mix ^1^	Continuous: 0 (single use) – 1 (perfect mixing)	National Spatial Data Infrastructure Portal [62]		0.50 (0.3)
Residential density ^1^	Continuous: Number of residential units per square kilometer	Statistical Geographic Information Service [73]		94.1 (24.9)
Public transport density ^1^	Continuous: Number of public transport destinations (bus stops and subway stations) per square kilometer	Seoul Transport Operation and Information Service [74], Road Name Address [61]		8.3 (2.2)

^1^ square root-transformed, SD: standard deviation.

**Table 3:** *t*-Test results of the comparison between areas with Walkability Score equal or greater than 50 (N = 395) and areas with Walkability Score less than 50 (N = 29).

Variables	Dongs with Walkability Score ≥50 (N = 395)		Dongs with Walkability Score <50 (N = 29)		*p*-Values for Difference	
	Mean	SD	Mean	SD	t	*p*-Value
Travel Mode						
Walking	55.4%	5.9%	50.5%	12.9%	2.1	0.049
Cycling	0.9%	1.0%	0.7%	1.0%	1.1	0.255
Public transport	30.7%	5.3%	32.2%	6.2%	−1.4	0.149
Private automobile	13.0%	6.4%	16.7%	14.8%	−1.3	0.196
Urban Forms						
Land use mix ^1^	0.5	0.3	0.6	0.1	−6.4	0.000
Residential density ^1^	94.7	25.2	55.1	16.8	11.8	0.000
Public transport density ^1^	8.4	2.2	5.9	1.7	7.8	0.000

^1^ square root-transformed.

**Table 4:** Multilevel logistic models for odds of non-motorized (walking and cycling) commuting.

Variables	Model 1				Model 2				Model 3			
	OR	*p*-Value	95% CI		OR	*p*-Value	95% CI		OR	*p*-Value	95% CI	
			Lower	Upper			Lower	Upper			Lower	Upper
Intercept.	3.090 ***	<0.001	3.026	3.154	2.511 ***	<0.001	2.358	2.664	2.387 ***	<0.001	2.218	2.557
Individual Variables (Level 1)												
Age	0.987 ***	<0.001	0.987	0.988	0.987 ***	<0.001	0.985	0.989	0.987 ***	<0.001	0.985	0.988
Gender (reference: male)	1.340 ***	<0.001	1.317	1.363	1.340 ***	<0.001	1.316	1.364	1.336 ***	<0.001	1.312	1.361
Income	0.932 ***	<0.001	0.919	0.945	0.936 ***	<0.001	0.922	0.950	0.936 ***	<0.001	0.922	0.951
Car ownership (reference: no)	0.713 ***	<0.001	0.684	0.742	0.707 ***	<0.001	0.674	0.740	0.707 ***	<0.001	0.673	0.741
Neighborhood Variables (Level 2; Dong level)												
Walkability Score					1.003 **	0.002	1.001	1.005	1.003 **	0.008	1.001	1.006
Land use mix ^1^									1.021	0.615	0.939	1.103
Residential density ^1^									0.999	0.136	0.998	1.000
Public transport density ^1^									1.012 *	0.031	1.001	1.023
AIC	173690.1				173218.1				170807.0			
BIC	173748.7				173423.3				171041.1			
ICC					2.1%				2.1%			
N	129,044											

*** *p* < 0.001, ** *p* < 0.01, * *p* < 0.05; ^1^ square root-transformed; OR = Odds Ratio; CI = Confidence Interval; AIC = Akaike Information Criterion; BIC = Bayesian Information Criterion; ICC = Intraclass Correlation Coefficient.
